# Association of SARS-CoV-2 Seropositivity and Symptomatic Reinfection in Children in Nicaragua

**DOI:** 10.1001/jamanetworkopen.2022.18794

**Published:** 2022-06-27

**Authors:** John Kubale, Angel Balmaseda, Aaron M. Frutos, Nery Sanchez, Miguel Plazaola, Sergio Ojeda, Saira Saborio, Roger Lopez, Carlos Barilla, Gerald Vasquez, Hanny Moreira, Anna Gajewski, Lora Campredon, Hannah E. Maier, Mahboob Chowdhury, Cristhiam Cerpas, Eva Harris, Guillermina Kuan, Aubree Gordon

**Affiliations:** 1 Department of Epidemiology, University of Michigan School of Public Health, Ann Arbor; 2 Centro Nacional de Diagnóstico y Referencia, Ministry of Health, Managua, Nicaragua; 3 Sustainable Sciences Institute, Managua, Nicaragua; 4 Centro de Salud Sócrates Flores Vivas, Ministry of Health, Managua, Nicaragua; 5 Division of Infectious Diseases and Vaccinology, School of Public Health, University of California, Berkeley

## Abstract

**Importance:**

The impact of the SARS-CoV-2 pandemic on children remains unclear. Better understanding of the burden of COVID-19 among children and their risk of reinfection is crucial, as they will be among the last groups vaccinated.

**Objective:**

To characterize the burden of COVID-19 and assess how risk of symptomatic reinfection may vary by age among children.

**Design, Setting, and Participants:**

In this prospective, community-based pediatric cohort study conducted from March 1, 2020, to October 15, 2021, 1964 nonimmunocompromised children aged 0 to 14 years were enrolled by random selection from the Nicaraguan Pediatric Influenza Cohort, a community-based cohort in District 2 of Managua, Nicaragua. Additional newborn infants aged 4 weeks or younger were randomly selected and enrolled monthly via home visits.

**Exposures:**

Prior COVID-19 infection as confirmed by positive anti–SARS-CoV-2 antibodies (receptor binding domain and spike protein) or real-time reverse transcriptase–polymerase chain reaction (RT-PCR)–confirmed COVID-19 infection at least 60 days before current COVID-19 infection.

**Main Outcomes and Measures:**

Symptomatic COVID-19 cases confirmed by real-time RT-PCR and hospitalization within 28 days of symptom onset of a confirmed COVID-19 case.

**Results:**

This cohort study assessed 1964 children (mean [SD] age, 6.9 [4.4] years; 985 [50.2%] male). Of 1824 children who were tested, 908 (49.8%; 95% CI, 47.5%-52.1%) were seropositive during the study. There were also 207 PCR-confirmed COVID-19 cases, 12 (5.8%) of which were severe enough to require hospitalization. Incidence of COVID-19 was highest among children younger than 2 years (16.1 cases per 100 person-years; 95% CI, 12.5-20.5 cases per 100 person-years), which was approximately 3 times the incidence rate in any other child age group assessed. In addition, 41 symptomatic SARS-CoV-2 episodes (19.8%; 95% CI, 14.4%-25.2%) were reinfections.

**Conclusions and Relevance:**

In this prospective, community-based pediatric cohort study, rates of symptomatic and severe COVID-19 were highest among the youngest participants, with rates stabilizing at approximately 5 years of age. In addition, symptomatic reinfections represented a large proportion of symptomatic COVID-19 cases.

## Key Points

**Question:**

What is the burden of COVID-19 among young children, and how does risk of reinfection vary with age?

**Findings:**

In this cohort study of 1964 children in Nicaragua aged 0 to 14 years, children younger than 2 years had the highest rates of symptomatic and severe COVID-19, particularly compared with children aged 5 to 14 years.

**Meaning:**

These findings suggest that the burden of COVID-19 and associated severe illness may not be evenly distributed across age groups in children.

## Teaser

This cohort study of children in Nicaragua assesses whether symptomatic reinfection with COVID-19 varies by age.

## Introduction

The SARS-CoV-2 pandemic has caused severe disease and death globally.^1,2^ Although most pediatric SARS-CoV-2 infections are mild or asymptomatic, severe illness can occur in children, including typical severe respiratory infections and multisystem inflammatory syndrome.^3,4^ Previous studies^5,6,7^ have suggested that younger children, older adolescents, and children with underlying health conditions more frequently present with symptomatic or severe COVID-19, although less commonly than adults. These groups may also be more likely to have long-lasting postacute sequelae, often referred to as long COVID, although still less likely than adults.^8,9^ However, most of the literature regarding the burden of SARS-CoV-2 in children comes from hospital-based studies,^4,6,10,11,12,13,14^ which underestimate the disease burden in children, particularly those with mild infections. The pediatric community-level evidence that does exist is largely from high-income countries and includes few children younger than 5 years.^15,16,17^ Despite understanding that differences in infection severity exist between adults and children, the burden and characteristics of SARS-CoV-2 infection among children remain poorly characterized.^3,18^

Understanding the implications of SARS-CoV-2 for children is particularly important because children will be among the last to be vaccinated. At the time of writing, only 1 vaccine has been approved in the US and Europe for children younger than 11 years,^19^ and vaccine supplies for many countries, particularly low- and middle-income countries, remain limited.^20^ In fact, as of November 12, 2021, only 6.5% of people in low-income countries have received at least 1 vaccine dose.^20,21^ However, even in high-income countries where older children are being vaccinated, it is critical to understand the natural history of SARS-CoV-2 in children.

As a consensus has grown around the likelihood of SARS-CoV-2 becoming endemic, researchers have begun exploring what the transition to endemicity might look like so that interventions might be tailored to be more effective. The strength and durability of immune protection against SARS-CoV-2 are perhaps the most important factors in this consideration. A February 2021 study by Lavine et al^22^ suggests that even though sterilizing immunity may wane quickly, if protection against severe infections remains relatively stable, SARS-CoV-2 may become no more severe than the known seasonal human coronaviruses. However, uncertainty regarding the natural history of SARS-CoV-2 infections limits our ability to determine whether this will occur. Of particular importance is the strength and durability of immune protection against illness after infection across the severity spectrum and whether the increased circulation of variants of concern has affected COVID-19 severity in children. These questions are also of great relevance to future emerging pathogens with pandemic potential. Using data from a community-based prospective pediatric cohort in Managua, Nicaragua, we aimed to assess the burden of infection and disease in this cohort and the risk of symptomatic reinfection.

## Methods

### Study Population and Sample Collection

Participants were from the Nicaraguan Pediatric Influenza Cohort, the methods of which have been described in detail previously.^23^ Briefly, from March 1, 2020, to October 15, 2021, children aged 0 to 14 years were enrolled when visiting the Health Center Sócrates Flores Vivas or through home visits and followed up until their 15th birthday or loss to follow-up. The study was approved by the institutional review boards of the Nicaraguan Ministry of Health and the University of Michigan. Written informed consent was obtained from a parent or guardian of all participants, and verbal assent was obtained from children 6 years or older. This study followed the Strengthening the Reporting of Observational Studies in Epidemiology (STROBE) reporting guideline.^24^

Parents agreed to bring their children to the study clinic (Health Center Sócrates Flores Vivas) for any illness, and all participants were provided with free medical care for the duration of their participation. Initially, respiratory samples were collected from participants aged 2 to 14 years presenting with fever or parent-reported fever and at least 1 of the following symptoms: cough, sore throat, or rhinorrhea. Respiratory samples were collected from participants younger than 2 years who presented with fever or parent-reported fever. In June 2020, these criteria were expanded so that samples were also collected from children who presented with loss of taste or smell, rash, conjunctivitis, or fever without a defined focus. Samples were collected using nasal and oropharyngeal flocked plastic swabs. Blood samples were obtained from children on enrollment and yearly thereafter (in February or March 2020 and February or March 2021). A random selection of children in the cohort in 2017 (n = 509) were enrolled into a household cohort study and had an additional blood sample collected in September or October 2020.

### Laboratory Methods

RNA was extracted from respiratory samples (QIAamp Viral RNA Mini Kit, Qiagen) and tested for SARS-CoV-2 via real-time reverse transcriptase–polymerase chain reaction (PCR).^25^ Blood samples were tested for antibodies against the SARS-CoV-2 receptor-binding domain and spike proteins via enzyme-linked immunosorbent assay (ELISA).^26^ Samples were tested for an end point using 4-fold dilution from 100 until 6400, and the titer for each sample was obtained using the Reed and Muench method.

### Data Collection and Case Definitions

Yearly surveys were administered at the individual and household levels every March or April, collecting data on a wide variety of social and environmental factors. A SARS-CoV-2–specific survey was also completed in September or October 2020 and February or March 2021. Finally, comprehensive medical consult forms were collected on each visit to the study health clinic along with data from follow-up visits conducted until the resolution of a child’s illness.

We classified the severity of COVID-19 as subclinical, mild, moderate, or severe based on criteria used in a household transmission study within the same community.^27^ These definitions differed from those of the National Institutes of Health in being less stringent in the classification of moderate or severe disease. Participants who reported no symptoms associated with their infection were classified as having subclinical illness. Those with any symptoms, excluding difficulty breathing, rapid breathing, or shortness of breath, were classified as having mild illness. Moderate COVID-19 was considered illness with associated difficulty breathing, rapid breathing, or shortness of breath. Finally, those requiring hospitalization were classified as having severe COVID-19. A more detailed description of the case definitions used in this analysis can be found in the eMethods in the Supplement.

Reinfection with SARS-CoV-2 was defined as a having a positive PCR result after an ELISA-positive result and/or a positive PCR result occurring at least 60 days after an earlier PCR-positive result. We considered PCR-positive results occurring within 59 days of each other to be from the same infection, because a previous study^28^ observed shedding detectable by PCR for longer than 30 days. For participants with positive ELISA results who did not have positive PCR results, we used surveys conducted in October or November 2020 and February or March 2021 to retrospectively assess COVID-19 illness severity. We observed almost no transmission of influenza or respiratory syncytial virus during periods of elevated SARS-CoV-2 transmission; thus, we assumed that symptoms reported by participants within these time periods were related to SARS-CoV-2. Symptoms reported between August 1, 2020, and February 15, 2021, were considered not related to SARS-CoV-2 unless there was a clear epidemiologic link (eg, confirmed infection in the household at the same time).

### Statistical Analysis

We calculated incidence rates for COVID-19 and COVID-19–associated hospitalization using a Poisson distribution to estimate 95% CIs.^29,30^ Risk of reinfection after SARS-CoV-2 infection was calculated for those with blood samples collected in October 2020 or later. Participants were considered seropositive starting the day their first ELISA-positive sample was collected. They were assumed to remain seropositive unless they had a subsequent sample that tested negative by ELISA. To compare against vaccine effectiveness, we estimated the protection from symptomatic reinfection as 1 − incidence rate ratio, hereafter referred to as protection. The incidence rate ratio was obtained by fitting age-stratified generalized linear models with a Poisson distribution. All analyses were conducted using R software, version 4.1.1 (R Foundation for Statistical Computing).

## Results

A total of 1964 children (mean [SD] age, 6.9 [4.4] years; 985 [50.2%] male and 979 [49.8%] female) participated in the study. Overall, participants contributed a total of 2706 person-years, a mean (SD) of 1.4 (0.4) years per person (Table 1). A total of 159 children (8.1%) withdrew from the study or were lost to follow-up. The most common reasons for early withdrawal were failure to appear for their yearly annual sample (70 [44.0%]) and inability to find the participant at home (44 [27.7%]). Participants recorded 9804 visits to the health center, with 1497 children (76.2%) having at least 1 visit. In all, 1014 participants (51.6%) were infected with SARS-CoV-2 as determined by PCR or ELISA during the study.

**Table 1.** Characteristics of Study Participants

Characteristic	Data (N = 1964)
Sex, No. (%)	
Male	985 (50.2)
Female	979 (49.8)
Age, mean (SD), y	6.9 (4.4)
Time in study, mean (SD), person-years	1.4 (0.4)
Water tap outside house, No. (%) (n = 1716)	584 (34.0)
No. of people in household, mean (SD) (n = 1716)	7.9 (4.4)

### Incidence of PCR-Confirmed COVID-19

**Figure 1.**
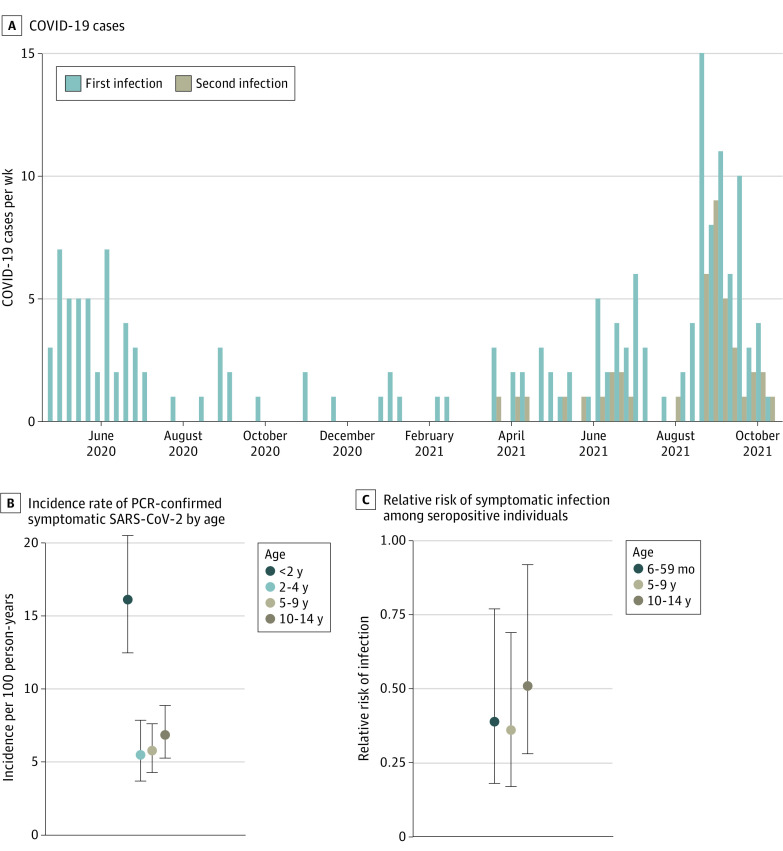
Symptomatic COVID-19 Infections in the Cohort PCR indicates polymerase chain reaction.

A total of 207 PCR-confirmed COVID-19 cases occurred in 201 participants (10.2%) (Table 2 and Figure 1A). Although low-level transmission occurred throughout the study, most cases occurred in 2 distinct waves: March to August 2020 and April to October 2021 (Figure 1A). The overall incidence rate of COVID-19 in the cohort was 7.7 (95% CI, 6.6-8.8) cases per 100 person-years. When examined by age, children younger than 2 years had the greatest incidence of COVID-19, with 16.1 (95% CI, 12.5-20.5) cases per 100 person-years (Table 2 and Figure 1B). In children older than 2 years, the incidence of COVID-19 was substantially lower but relatively stable, with rates of 5.5 cases (95% CI, 3.7-7.9) per 100 person-years among children aged 2 to 4 years, 5.8 cases (95% CI, 4.3-7.6) per 100 person-years among children aged 5 to 9 years, and 6.9 cases (95% CI, 5.3-8.9) per 100 person-years among children aged 10 to 14 years (Table 2 and Figure 1B). A slightly higher incidence of COVID-19 was found in female participants (8.4 [95% CI, 7.0-10.1] cases per 100 person-years) compared with male participants (6.8 [95% CI, 5.6-8.4] cases per 100 person-years); however, the difference was not significant (Table 2).

**Table 2.** Incidence of Symptomatic SARS-CoV-2

Characteristic	No. (%) of participants	No. of person-years	Incidence rate per 100 person-years (95% CI)
Total	207	2706	7.7 (6.6-8.8)
Sex			
Male	93 (44.9)	1352	6.8 (5.6-8.4)
Female	114 (55.1)	1354	8.4 (7.0-10.1)
Age, y			
<2	66 (31.9)	410	16.1 (12.5-20.5)
2-4	30 (14.5)	543	5.5 (3.7-7.9)
5-9	52 (25.1)	897	5.8 (4.3-7.6)
10-14	59 (28.5)	856	6.9 (5.3-8.9)

### ELISA-Confirmed Symptomatic SARS-CoV-2 Infection

**Figure 2.**
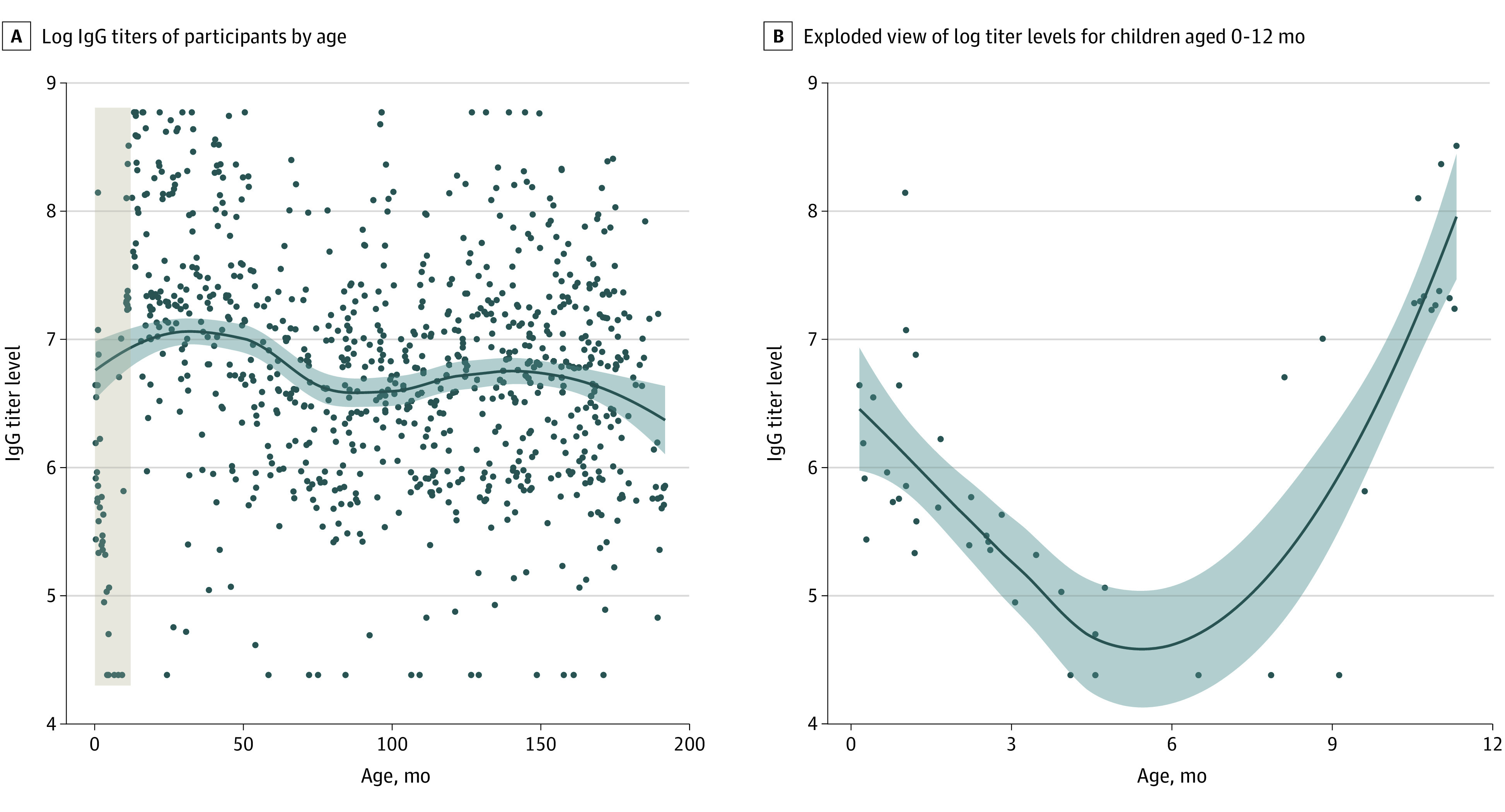
Anti–SARS-CoV-2 IgG Titers by Age A, Brown shaded area presented in exploded view in panel B. Data underwent LOESS (locally estimated scatterplot smoothing). Shaded areas indicate 95% CIs.

ELISA results for anti–SARS-CoV-2 antibodies were obtained for 1824 participants (92.9%), with 908 (49.8%; 95% CI, 47.5%-52.1%) having a seropositive status throughout the study. Antibody titers were obtained from 877 participants (96.6%) with a seropositive status. Seroreversion was rare, with 11 participants (1.2%) with a seropositive status subsequently testing negative by ELISA. Of these, 10 (90.9%) were older than 5 years, whereas 1 was younger than 6 months, suggesting a possible loss of maternal antibodies. In addition, among children who seroreverted, only 2 had experienced illness episodes that were symptomatic (both were mild); the rest were subclinical. Titers were highest among young children, decreasing between 4 and 6 years of age before reaching a plateau, where they remained relatively stable (Figure 2A). We saw evidence of waning maternal antibodies among young infants, with titers steadily decreasing in the first 4 months of life before beginning to increase again (Figure 2B). We observed no meaningful difference in titers by sex (eFigure 1 in the Supplement).

### COVID-19 Severity

Among PCR-confirmed COVID-19 cases, 12 (5.8%) required hospitalization, resulting in an incidence rate of 0.4 (95% CI, 0.2-0.8) per 100 person-years (eTable 1 in the Supplement). Children younger than 2 years had the highest rate of hospitalization associated with COVID-19—2.8 times that of children aged 2 to 4 years and 8.5 times that of children aged 5 to 9 years, although only the differences between those younger than 2 years (1.7 [95% CI, 0.7-3.5] cases per 100 person-years) and those 5 to 9 years (0.2 [95% CI, 0.03-0.8] cases per 100 person-years) were statistically significant (eTable 1 and eFigure 2A in the Supplement). We did not observe any children aged 10 to 14 years with COVID-19 who required hospitalization. We did not detect a statistically significant difference by sex (eFigure 2B in the Supplement).

### Clinical Presentation and Post-COVID Sequelae

Children with PCR-confirmed COVID-19 presented with a variety of symptoms, with runny nose (155 [74.9%]), cough (148 [71.5%]), and fever or feverishness (139 [67.1%]) being the most common (eTable 2 and eFigure 2 in the Supplement). Although symptoms resolved within 4 weeks for most children with PCR-confirmed infections, 21 participants (10.4%) had at least 1 symptom that was present for 28 days or more. Of these, 9 (13.6%) were younger than 2 years, 2 (6.7%) were aged 2 to 4 years, 3 (5.8%) were aged 5 to 9 years, and 7 (11.9%) were aged 10 to 14 years. Most participants with longer-lasting sequelae (≥28 days) had upper respiratory tract symptoms (14 with runny nose, 7 with cough, 3 with sore throat, and 3 with nasal congestion), whereas few had long-duration nonspecific symptoms (4 with fever and 5 with a headache). One participant younger than 2 years still had diarrhea, and another participant younger than 2 years had rapid breathing.

### Risk of Symptomatic Second SARS-CoV-2 Infection

Symptomatic reinfection with SARS-CoV-2 was relatively common in the cohort, with 41 PCR-confirmed episodes (19.8%; 95% CI, 14.4%-25.2%) occurring in children with previous SARS-CoV-2 infection detected by PCR or ELISA. All second infections occurred in 2021, when variants of concern, particularly Gamma and Delta, began actively circulating in Nicaragua.^31^ The relative rate (RR) of symptomatic second infection followed a similar pattern to that observed with all COVID-19 cases and associated severe presentations, such as hospitalization. Children 6 months to 4 years of age displayed slightly higher protection from symptomatic second infection at 61% (RR, 0.39; 95% CI, 0.18-0.77) as did children 5 to 9 years of age at 64% (RR, 0.36; 0.17-0.69) compared with 49% (RR, 0.51; 0.28-0.92) among children 10 to 14 years of age, although these differences were not statistically significant (Table 3 and Figure 1C). Children younger than 6 months at ELISA testing were excluded from this analysis to rule out potential bias from maternal antibodies. The importance of maternal antibodies among children younger than 6 months was confirmed by the antibody titers we observed in the first year of life, a steady decrease in titer levels in the first 6 months followed by an increase from 6 to 12 months (Figure 2B). Although moderate and severe secondary cases of COVID-19 occurred, we did not detect any difference in severity between the first and second infections (eTable 3 in the Supplement).

**Table 3.** Protection From Symptomatic Reinfection

Characteristic	No. of second infections (n = 41), No. (%)	Protection^a^	
		Rate, %	RR (95% CI)
Sex			
Male	14 (34.1)	62	0.38 (0.20-0.69)
Female	27 (65.9)	55	0.45 (0.28-0.71)
Age			
6-59 mo	10 (24.4)	61	0.39 (0.18-0.77)
5-9 y	11 (26.8)	64	0.36 (0.17-0.69)
10-14 y	20 (48.8)	49	0.51 (0.28-0.92)

Abbreviation: RR, relative rate.

^a^ Protection calculated as 1 − RR.

## Discussion

In this cohort study, we assessed the incidence and severity of SARS-CoV-2 infections, as well as the risk of symptomatic reinfection, within a prospective, community-based cohort of children in Nicaragua. We observed high rates of infection, with 51.6% of children being infected during the study. Most COVID-19 episodes were mild or subclinical (eTable 4 in the Supplement), but severe illness requiring hospitalization occurred, particularly among children younger than 2 years (eTable 1 in the Supplement). Symptomatic reinfection with SARS-CoV-2 was also common, with 19.8% (95% CI, 14.4%-25.2%) of symptomatic COVID-19 episodes being second infections. This finding contributed to the greater levels of risk for symptomatic re-infection that we observed compared to an earlier study in the same community.^27^

Our findings regarding the relative frequency and severity of COVID-19 in children are consistent with those in the literature, specifically that pediatric SARS-CoV-2 infections are common but largely mild or subclinical.^5,6,7^ Another study^32^ in Nicaragua reported that 37.7% of children younger than 15 years were seropositive for SARS-CoV-2, which is substantially lower than the 49.8% (95% CI, 47.5%-52.1%) we observed in our study. This rate was, however, consistent with that estimated from a previously published analysis^27^ in a household transmission cohort in the same community where 56.7% of participants were infected. Although more than 50% of the cohort were infected during the study, only 207 cases (19.6%) were detected by PCR, which again demonstrates that symptomatic COVID-19 cases make up only a small proportion of infections in children and underscores the importance of community-based, prospective studies in accurately characterizing the natural history of pediatric SARS-CoV-2 infection. Previous studies^9,15^ have also indicated that although most children with symptomatic infections observe symptom resolution within a few weeks, some children experience sustained symptoms. Among children with symptomatic SARS-CoV-2 infection, we observed 21 (10.1%; 95% CI, 6.0%-14.3%) with symptoms that were present for 28 days or longer after symptom onset, a larger proportion than the 4.4% reported by Molteni et al.^15^ However, this difference can likely be attributed to their not including children younger than 5 years, because the youngest and oldest age groups in our study displayed the highest frequency of symptoms lasting 28 days or longer. The most common symptoms that persisted beyond 28 days of onset in our study also differed from previous reports,^9,15^ with upper respiratory tract symptoms being the most common rather than the more nonspecific symptoms of fatigue and headache reported by others.

Given the frequently mild presentation of pediatric SARS-CoV-2 infections, the relative dearth of community-based cohort studies of SARS-CoV-2 infection in children has resulted in a poor understanding of the risk of reinfection. A previous study^27^ in this same community found that risk after infection was similar to that after vaccination; however, this study was performed before variants of concern were widely circulating in Nicaragua and was not powered to assess risk of reinfection among young children. In the current study, we found that symptomatic second infections were common, making up approximately 20% of all PCR-confirmed episodes of symptomatic SARS-CoV-2 infection, with children 6 to 59 months and 5 to 9 years displaying lower risk of symptomatic second infection (Table 3, Figure 1C), although these differences were not statistically significant. Notably, some of these second infections resulted in illnesses classified as moderate or severe. We were underpowered to sufficiently explore severity of second infections compared with first infections. Given its implications for pediatric vaccination strategies, the relative severity of second SARS-CoV-2 infections in children should be monitored closely moving forward.^22^

### Strengths and Limitations

This study has several strengths. First, as a prospective, community-based pediatric cohort study, it provides valuable insight into the burden of symptomatic SARS-CoV-2 infection within a population that is likely to be among the last to be vaccinated—children in low- and middle-income countries. Second, by using well-established systems for sample collection and testing combined with robust data collection, we were able to characterize key aspects of SARS-CoV-2 presentation and severity that remain poorly described among children. Third, by incorporating serologic testing and genetic sequencing, we were able to estimate the risk of reinfection in the context of variants such as Gamma and Delta. Fourth, although durability of immunity against SARS-CoV-2 infection after vaccination is being widely studied, fewer data are available regarding immunity after SARS-CoV-2 infection, particularly among children.

This analysis also has several limitations. The initial serologic samples were collected in February or March 2020, largely before the first wave of SARS-CoV-2 infections in Nicaragua. In the absence of a laboratory test, we also required an epidemiologic link for those reporting symptoms between August 1, 2020, and February 15, 2021, for an illness to be considered SARS-CoV-2 related. As such, it is possible that some infections, particularly milder presentations or those with less common symptom presentations (eg, gastrointestinal symptoms), were missed; however, given that a large respiratory syncytial virus epidemic occurred during that time, our definition leads to the least misclassification. In addition, most of these infections were captured via PCR or ELISA testing of samples collected in late 2020 and early 2021. To assess illness severity of infections detected only by ELISA, we relied on retrospective surveys that may have been subject to recall bias and limited our ability to pinpoint the exact time of infection for many mild cases. Fortunately, this likely affected only the mildest of cases given the expansive PCR testing criteria used. The initial testing criteria (before June 2020) were slightly broader for children younger than 2 years, which could have biased our estimates of the incidence of symptomatic COVID-19 by age. However, very few children younger than 2 years were identified using these broader criteria, so we would expect any bias to be small. As a community-based, prospective cohort study, this study was underpowered to assess the burden of multisystem inflammatory syndrome and death associated with SARS-CoV-2 infection because of their rarity. Finally, this study was conducted before the introduction of the Omicron variant to the population.

## Conclusions

In this prospective cohort study of children in Nicaragua, we were able to explore SARS-CoV-2 infections in children aged 0 to 14 years at the community level. We observed high rates of infection, with 51.6% of children having been infected with SARS-CoV-2 during the study. Although illness was generally mild, severe illness and prolonged sequelae were observed—most often among children younger than 2 years. Finally, symptomatic reinfection was common, highlighting the importance of waning immunity after infection in children.

## Notes

Supplement. **eMethods.** Case Definitions **eTable 1.** Incidence of COVID-19 Associated Hospitalization **eTable 2.** Symptom Presentation of COVID-19 Cases by Age **eTable 3.** Risk of Moderate or Severe COVID-19 by Sero-status **eTable 4.** SARS-CoV-2 Illness Severity **eFigure 1.** IgG Titers by Sex **eFigure 2.** Incidence Rate Ratios of COVID-19 Associated Hospitalization

## References

[1] O’Driscoll M, Ribeiro Dos Santos G, Wang L, . Age-specific mortality and immunity patterns of SARS-CoV-2. Nature. 2021;590(7844):140-145. doi:10.1038/s41586-020-2918-0 33137809

[2] Parohan M, Yaghoubi S, Seraji A, Javanbakht MH, Sarraf P, Djalali M. Risk factors for mortality in patients with coronavirus disease 2019 (COVID-19) infection: a systematic review and meta-analysis of observational studies. Aging Male. 2020;23(5):1416-1424. doi:10.1080/13685538.2020.1774748 32508193

[3] Bhuiyan MU, Stiboy E, Hassan MZ, . Epidemiology of COVID-19 infection in young children under five years: a systematic review and meta-analysis. Vaccine. 2021;39(4):667-677. doi:10.1016/j.vaccine.2020.11.078 33342635 PMC7833125

[4] Parcha V, Booker KS, Kalra R, . A retrospective cohort study of 12,306 pediatric COVID-19 patients in the United States. Sci Rep. 2021;11(1):10231. doi:10.1038/s41598-021-89553-1 33986390 PMC8119690

[5] Kufa T, Jassat W, Cohen C, . Epidemiology of SARS-CoV-2 infection and SARS-CoV-2 positive hospital admissions among children in South Africa. Influenza Other Respir Viruses. 2022;16(1):34-47. doi:10.1111/irv.12916 34796674 PMC9664941

[6] Bailey LC, Razzaghi H, Burrows EK, . Assessment of 135 794 pediatric patients tested for severe acute respiratory syndrome coronavirus 2 across the United States. JAMA Pediatr. 2021;175(2):176-184. doi:10.1001/jamapediatrics.2020.5052 33226415 PMC7684518

[7] Bolaños-Almeida CE, Espitia Segura OM. Clinical and epidemiologic analysis of COVID-19 children cases in Colombia PEDIACOVID. Pediatr Infect Dis J. 2021;40(1):e7-e11. doi:10.1097/INF.0000000000002952 33093428

[8] Smane L, Roge I, Pucuka Z, Pavare J. Clinical features of pediatric post-acute COVID-19: a descriptive retrospective follow-up study. Ital J Pediatr. 2021;47(1):177. doi:10.1186/s13052-021-01127-z 34446085 PMC8390049

[9] Brackel CLH, Lap CR, Buddingh EP, . Pediatric long-COVID: an overlooked phenomenon? Pediatr Pulmonol. 2021;56(8):2495-2502. doi:10.1002/ppul.25521 34102037 PMC8242715

[10] Götzinger F, Santiago-García B, Noguera-Julián A, ; ptbnet COVID-19 Study Group. COVID-19 in children and adolescents in Europe: a multinational, multicentre cohort study. Lancet Child Adolesc Health. 2020;4(9):653-661. doi:10.1016/S2352-4642(20)30177-2 32593339 PMC7316447

[11] Loomba RS, Villarreal EG, Farias JS, Bronicki RA, Flores S. Pediatric intensive care unit admissions for COVID-19: insights using state-level data. Int J Pediatr. 2020;2020:9680905. doi:10.1155/2020/9680905 33299428 PMC7704189

[12] Salako A, Odubela O, Musari-Martins T, . Prevalence and presentation of paediatric coronavirus disease 2019 in Lagos, Nigeria. Int J Pediatr. 2021;2021:2185161. doi:10.1155/2021/2185161 34659422 PMC8514970

[13] Saleh NY, Aboelghar HM, Salem SS, . The severity and atypical presentations of COVID-19 infection in pediatrics. BMC Pediatr. 2021;21(1):144. doi:10.1186/s12887-021-02614-2 33765980 PMC7992820

[14] Somekh I, Stein M, Karakis I, Simões EAF, Somekh E. Characteristics of SARS-CoV-2 infections in Israeli children during the circulation of different SARS-CoV-2 variants. JAMA Netw Open. 2021;4(9):e2124343. doi:10.1001/jamanetworkopen.2021.24343 34491353 PMC8424472

[15] Molteni E, Sudre CH, Canas LS, . Illness duration and symptom profile in symptomatic UK school-aged children tested for SARS-CoV-2. Lancet Child Adolesc Health. 2021;5(10):708-718. doi:10.1016/S2352-4642(21)00198-X 34358472 PMC8443448

[16] Waterfield T, Watson C, Moore R, . Seroprevalence of SARS-CoV-2 antibodies in children: a prospective multicentre cohort study. Arch Dis Child. 2021;106(7):680-686. doi:10.1136/archdischild-2020-320558 33172887

[17] Watson AM, Haraldsdottir K, Biese KM, Goodavish L, Stevens B, McGuine TA. COVID-19 in US youth soccer athletes during summer 2020. J Athl Train. 2021;56(6):542-547. doi:10.4085/610-20 34375980 PMC8223610

[18] Wiedenmann M, Goutaki M, Keiser O, Stringhini S, Tanner M, Low N. The role of children and adolescents in the SARS-CoV-2 pandemic: a rapid review. Swiss Med Wkly. 2021;151:w30058. 34546012 10.4414/smw.2021.w30058

[19] Pfizer BioNTech. Pfizer and BioNTech Announce Positive Topline Results From Pivotal Trial of COVID-19 Vaccine in Children 5 to 11 Years. Updated September 20, 2021. Accessed November 7, 2021. https://www.businesswire.com/news/home/20210920005452/en/

[20] Ritchie H, Mathieu E, Rodés-Guirao L, Appel C, Giattino C, Ortiz-Ospina E, Hasell J, Macdonald B, Beltekian D, Roser M. Coronavirus Pandemic (COVID-19): Our World in Data. 2020. Accessed May 17, 2020. https://ourworldindata.org/coronavirus

[21] UNDP. Global Dashboard of Vaccine Equity. 2021. Accessed November 1, 2021. https://data.undp.org/vaccine-equity/

[22] Lavine JS, Bjornstad ON, Antia R. Immunological characteristics govern the transition of COVID-19 to endemicity. Science. 2021;371(6530):741-745. doi:10.1126/science.abe6522 33436525 PMC7932103

[23] Gordon A, Kuan G, Aviles W, . The Nicaraguan pediatric influenza cohort study: design, methods, use of technology, and compliance. BMC Infect Dis. 2015;15:504. doi:10.1186/s12879-015-1256-6 26553094 PMC4640204

[24] von Elm E, Altman DG, Egger M, Pocock SJ, Gøtzsche PC, Vandenbroucke JP; STROBE Initiative. The Strengthening the Reporting of Observational Studies in Epidemiology (STROBE) statement: guidelines for reporting observational studies. PLoS Med. 2007;4(10):e296. doi:10.1371/journal.pmed.0040296 17941714 PMC2020495

[25] Chu DKW, Pan Y, Cheng SMS, . Molecular diagnosis of a novel coronavirus (2019-nCoV) causing an outbreak of pneumonia. Clin Chem. 2020;66(4):549-555. doi:10.1093/clinchem/hvaa029 32031583 PMC7108203

[26] Amanat F, Stadlbauer D, Strohmeier S, . A serological assay to detect SARS-CoV-2 seroconversion in humans. medRxiv. Preprint posted online April 16, 2020. doi:10.1101/2020.03.17.20037713 PMC8183627 32398876

[27] Maier HE, Kuan G, Saborio S, . Clinical spectrum of SARS-CoV-2 infection and protection from symptomatic re-infection. Clin Infect Dis. 2021;ciab717. doi:10.1093/cid/ciab717 34411230 PMC8499752

[28] van Kampen JJA, van de Vijver DAMC, Fraaij PLA, . Duration and key determinants of infectious virus shedding in hospitalized patients with coronavirus disease-2019 (COVID-19). Nat Commun. 2021;12(1):267. doi:10.1038/s41467-020-20568-4 33431879 PMC7801729

[29] Stevenson M, Nunes T, Sanchez J, . *EpiR: An R Package for the Analysis of Epidemiological Data*. R Foundation for Statistical Computing; 2013:9-43.

[30] Ulm K. A simple method to calculate the confidence interval of a standardized mortality ratio (SMR). Am J Epidemiol. 1990;131(2):373-375. doi:10.1093/oxfordjournals.aje.a115507 2296988

[31] Maier HE, Balmaseda A, Ojeda S, . An immune correlate of SARS-CoV-2 infection and severity of reinfections. medRxiv. Preprint posted online November 24, 2021. doi:10.1101/2021.11.23.21266767

[32] González F, Vielot NA, Sciaudone M, . Seroepidemiology of SARS-CoV-2 infections in an urban Nicaraguan population. medRxiv. Preprint posted online March 1, 2021. doi:10.1101/2021.02.25.21252447 PMC8674192 35172912

