# Supplementary material for: Association of SARS-CoV-2 Seropositivity and Symptomatic Reinfection in Children in Nicaragua
Source: JAMA Netw Open. 2022 Jun 27;5(6):e2218794. doi: 10.1001/jamanetworkopen.2022.18794 (PMC9237791; doi:10.1001/jamanetworkopen.2022.18794)
Supplement: Supplement. — eMethods. Case Definitions eTable 1. Incidence of COVID-19 Associated Hospitalization eTable 2. Symptom Presentation of COVID-19 Cases by Age eTable 3. Risk of Moderate or Severe COVID-19 by Sero-status eTable 4. SARS-CoV-2 Illness Severity eFigure 1. IgG Titers by Sex eFigure 2. Incidence Rate Ratios of COVID-19 Associated Hospitalization [file jamanetwopen-e2218794-s001.pdf]

Supplementary Online Content

Kubale J, Balmaseda A, Frutos AM, et al. Association of SARS-CoV-2 seropositivity and symptomatic reinfection in children in Nicaragua. *JAMA Netw Open*. 2022;5(6):e2218794. doi:10.1001/jamanetworkopen.2022.18794

eMethods. Case Definitions

eTable 1. Incidence of COVID-19 Associated Hospitalization

eTable 2. Symptom Presentation of COVID-19 Cases by Age

eTable 3. Risk of Moderate or Severe COVID-19 by Sero-status

eTable 4. SARS-CoV-2 Illness Severity

eFigure 1. IgG Titers by Sex

eFigure 2. Incidence Rate Ratios of COVID-19 Associated Hospitalization

This supplementary material has been provided by the authors to give readers additional information about their work.

© 2022 Kubale J et al. *JAMA Network Open.*

eMethods. Case Definitions

Term		Definition
COVID-19 case		RT-PCR positive for SARS-CoV-2 and no prior positive within previous 59 days
Primary episode		RT-PCR-confirmed case of COVID-19 in participant with no prior positive via PCR or ELISA<br>OR<br>RT-PCR-confirmed case of COVID-19 in participant with prior ELISA positive<br>AND<br>age <6 months at time of ELISA positive
Secondary episode		RT-PCR-confirmed case of COVID-19 in participant with prior ELISA positive<br>AND<br>age ≥ 6 months at time of ELISA positive<br>OR<br>RT-PCR-confirmed case of COVID-19 in participant with prior RT-PCR-confirmed case of COVID-19 occurring >59 days prior.
Severity	Subclinical	No reported symptoms
	Mild	1 or more of the following symptoms:<br>• loss of taste<br>• loss of smell<br>• runny nose<br>• cough<br>• headache<br>• sore throat<br>• joint pain<br>• fever<br>• muscle pain<br>• diarrhea<br>• fatigue<br>• rash<br>• stayed in bed<br>• conjunctivitis<br>• congestion<br>• itchy throat<br>• loss of appetite<br>• faintness<br>• tight chest<br>• chest pain
	Moderate	Any of the following symptoms:<br>• difficulty breathing<br>• rapid breathing<br>• shortness of breath
	Severe	Transfer to hospital
Long COVID		Participants with any symptom persisting for at least 28 days following illness onset for confirmed COVID-19

© 2022 Kubale J et al. *JAMA Network Open.*

eTable 1. Incidence of COVID-19 Associated Hospitalization

**Characteristic**		**N(%)=12**	**Incidence Rate per 100 person-years**
Male		7(58.3)	0.5 (0.2-1.1)
Female		5(41.7)	0.4 (0.1-0.9)
Age	<2 years	7(58.3)	1.7 (0.7-3.5)
	2-4 years	3(25.0)	0.6 (0.1-1.6)
	5-9 years	2(16.7)	0.2 (0.03-0.8)
	10-14 years	0	--
[a]95% confidence intervals calculated using Poisson distribution.			

© 2022 Kubale J et al. *JAMA Network Open.*

eTable 2. Symptom Presentation of COVID-19 Cases by Age

Characteristic	<2 years, N = 66[a]	2-4 years, N = 30[a]	5-9 years, N = 52[a]	10-14 years, N = 59[a]	Overall, N = 207[a]
Loss of taste	0 (0)	0 (0)	3 (5.8)	8 (14)	11 (5.3)
Loss of smell	0 (0)	0 (0)	3 (5.8)	11 (19)	14 (6.8)
Runny nose	48 (73)	26 (87)	37 (71)	44 (75)	155 (75)
Cough	48 (73)	23 (77)	36 (69)	41 (69)	148 (71)
Headache	0 (0)	6 (20)	24 (46)	37 (63)	67 (32)
Sore throat	3 (4.5)	7 (23)	26 (50)	28 (47)	64 (31)
Fever/feverish	46 (70)	20 (67)	35 (67)	38 (64)	139 (67)
Joint pain	0 (0)	1 (3.3)	3 (5.8)	14 (24)	18 (8.7)
Muscle pain	0 (0)	2 (6.7)	6 (12)	15 (25)	23 (11)
Diarrhea	19 (29)	3 (10)	10 (19)	6 (10)	38 (18)
Vomiting	5 (7.6)	0 (0)	4 (7.7)	3 (5.1)	12 (5.8)
Fatigue	0 (0)	0 (0)	0 (0)	1 (1.7)	1 (0.5)
Rash	1 (1.5)	1 (3.3)	0 (0)	1 (1.7)	3 (1.4)
Stayed in bed	0 (0)	0 (0)	1 (1.9)	1 (1.7)	2 (1.0)
Conjunctivitis	0 (0)	0 (0)	0 (0)	0 (0)	0 (0)
Congestion	30 (45)	14 (47)	23 (44)	23 (39)	90 (43)
Itchy throat	1 (1.5)	0 (0)	3 (5.8)	5 (8.5)	9 (4.3)
Loss of appetite	15 (23)	11 (37)	17 (33)	7 (12)	50 (24)
Fainted	0 (0)	0 (0)	0 (0)	0 (0)	0 (0)
Difficulty breathing	3 (4.5)	2 (6.7)	4 (7.7)	3 (5.1)	12 (5.8)
Rapid breathing	4 (6.1)	1 (3.3)	2 (3.8)	0 (0)	7 (3.4)
Shortness of breath	0 (0)	0 (0)	1 (1.9)	2 (3.4)	3 (1.4)
Chest tightness	1 (1.5)	0 (0)	0 (0)	1 (1.7)	2 (1.0)
Chest pain	0 (0)	0 (0)	0 (0)	3 (5.1)	3 (1.4)
[a]N(%)					

© 2022 Kubale J et al. *JAMA Network Open.*

eTable 3. Risk of Moderate or Severe COVID-19 by Serostatus

	N=10	Person-years	IRR[a]
Seropositive	5	578.2	0.9 (0.3-3.2)
Seronegative	5	515.1	--
[a]Incidence Rate Ratio (95% confidence interval)			

eTable 4. SARS-CoV-2 Illness Severity

Characteristic		Subclinical	Mild	Moderate	Severe	Total
Female		409 (73.6)	131 (23.6)	11 (2.0)	5 (0.9)	556
Male		382 (74.5)	112 (21.8)	12 (2.3)	7 (1.4)	513
Age	<2 years	128 (65.3)	58 (29.6)	3 (1.5)	7 (3.6)	196
	2-4 years	148 (79.6)	32 (17.2)	3 (1.6)	3 (1.6)	186
	5-9 years	254 (77.2)	65 (19.8)	8 (2.4)	2 (0.6)	329
	10-14 years	261 (72.9)	88 (24.6)	9 (2.5)	0 (0.0)	358
Total		791 (74.0)	243 (22.7)	23 (2.2)	12 (1.1)	1069
[1] Values correspond to N(%)						

© 2022 Kubale J et al. *JAMA Network Open.*

eFigure 1. Anti SARS-CoV-2 IgG Titers by Sex

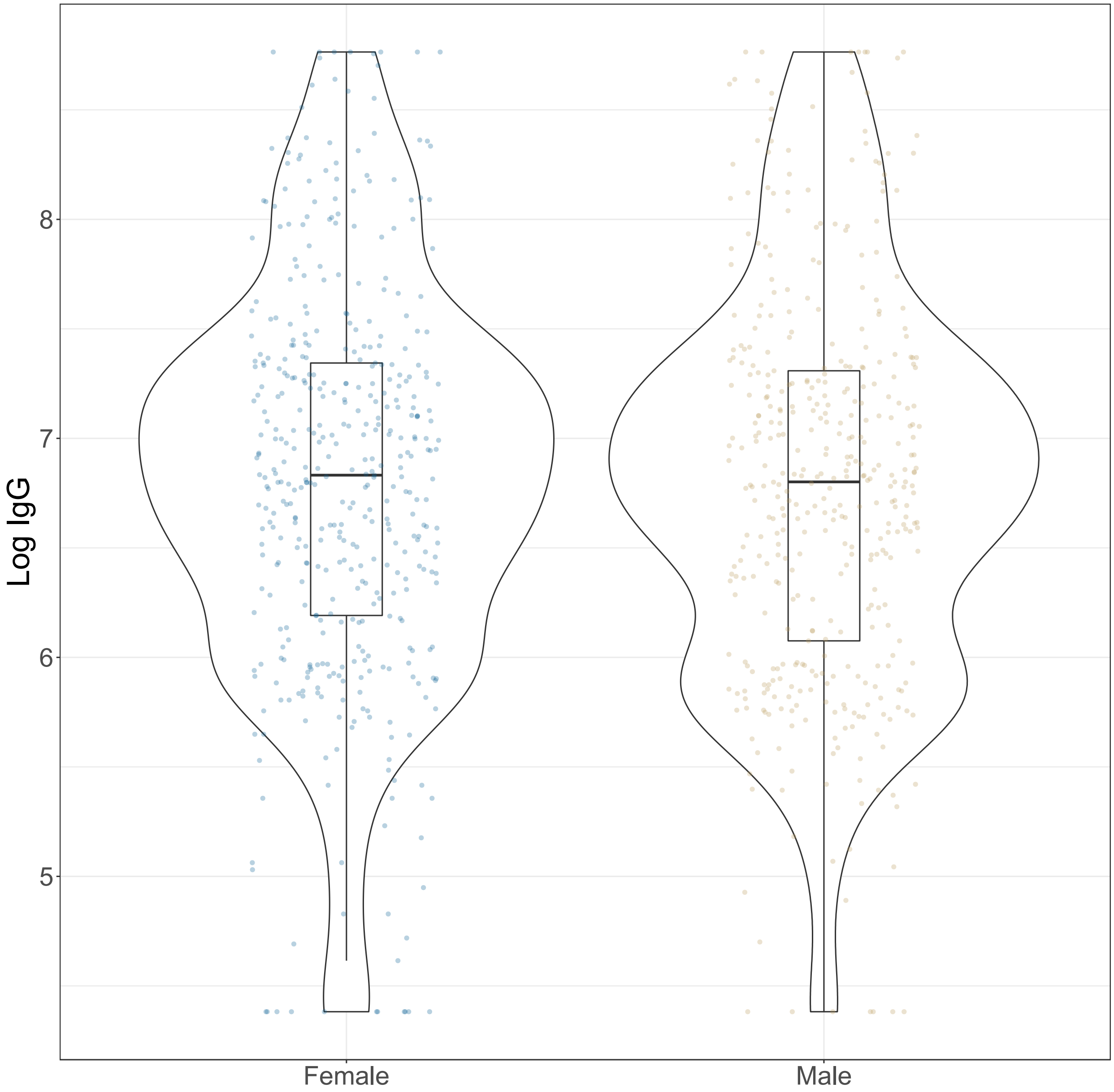

© 2022 Kubale J et al. *JAMA Network Open.*

eFigure 2. Incidence Rate Ratios of COVID-19 Associated Hospitalization

Panel A shows the incidence rate ratios of COVID-19 associated hospitalization and associated 95% confidence intervals (CI) calculated using a generalized linear model (GLM) with a Poisson distribution and a categorical age variable as the predictor. We observed no COVID-19 associated hospitalizations in those aged 10-14 years so they were not included in the figure. Panel B shows the incidence rate ratio of COVID-19 associated hospitalizations and 95% CI comparing females to males using a GLM with a Poisson distribution and a categorical sex variable as the predictor.

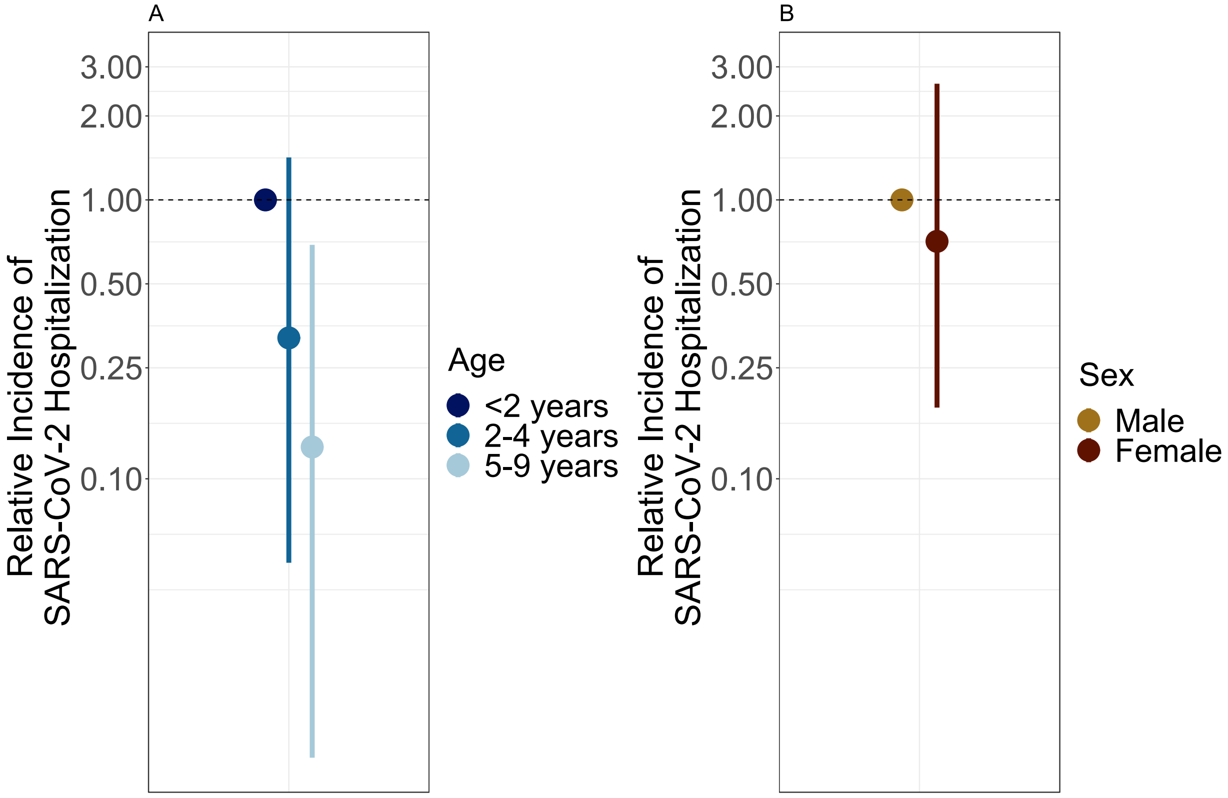

© 2022 Kubale J et al. *JAMA Network Open.*
